# The mechanism by which Naru 3 pill protects against intervertebral disc cartilage endplate degeneration based on network pharmacology and experimental verification

**DOI:** 10.1186/s13018-023-04014-x

**Published:** 2023-07-31

**Authors:** Jialin Guo, Jianmin Xue, Zhiwei He, Haiyu Jia, Xuejun Yang

**Affiliations:** 1 Inner Mongolia Medical University, Hohhot, 010050 Inner Mongolia China; 2 The Second Affiliated Hospital of Inner Mongolia Medical University, Hohhot, 010010 Inner Mongolia China; 3 The Affiliated Hospital of Inner Mongolia Medical University, NO.1 North Tongdao Road, Hohhot, 010030 Inner Mongolia China; 4 Peking University Cancer Hospital (Inner Mongolia Campus)/Affiliated Cancer Hospital of Inner Mongolia Medical University, NO.42 Zhaowuda Road, Hohhot, 010010 Inner Mongolia China

**Keywords:** Intervertebral disc degeneration, Naru 3 pill, Sesamin, Pharmacological mechanisms, Apoptosis

## Abstract

**Context:**

Naru 3 pill is a traditional Mongolian medicine for the treatment of intervertebral disc degeneration (IDD), but the mechanism is not yet clear.

**Objective:**

This study investigated the mechanism of Naru 3 pill in the treatment of IDD.

**Materials and methods:**

Active ingredients and related targets of Naru 3 pill, as well as IDD-related genes, were collected from public databases. The analysis was performed by protein‒protein interaction network analysis, gene ontology and Kyoto Gene and Genome Encyclopedia (KEGG) functional enrichment analysis, molecular docking and molecular dynamics simulations. Finally, the network pharmacology results were validated by in vitro experiments.

**Results:**

Network analysis showed that sesamin, piperine and ellagic acid were potential key components and CASP3, BAX and BCL2 were key targets. KEGG analysis indicated the apoptotic pathway as a potential pathway. Molecular docking showed that sesamin interacted better with the targets than the other components. The results of molecular dynamics simulations indicated that the three systems BAX-sesamin, BCL2-sesamin and CASP3-sesamin were stable and reasonable during the simulation. In vitro experiments showed that sesamin had the least effect on cell growth and the most pronounced proliferation-promoting effect, and so sesamin was considered the key component. The experiments confirmed that sesamin had antiapoptotic effects and reversed the expression of CASP3, BAX and BCL2 in degeneration models, which was consistent with the network pharmacology results. Furthermore, sesamin alleviated extracellular matrix (ECM) degeneration and promoted cell proliferation in the IDD model.

**Conclusion:**

The present study suggested that Naru 3 pill might exert its therapeutic and antiapoptotic effects on IDD by delaying ECM degradation and promoting cell proliferation, which provides a new strategy for the treatment of IDD.

**Supplementary Information:**

The online version contains supplementary material available at 10.1186/s13018-023-04014-x.

## Introduction

Low back pain (LBP) is a common clinical condition that has a severe impact on the lives and work of 70–85% of the world’s population [1]. Intervertebral disc degeneration (IDD), which is the causative agent of diseases such as disc herniation and lumbar stenosis, is considered a major cause of LBP and places a heavy burden on the global health care system [2]. The standard intervertebral disc (IVD) is composed of an intermediate nucleus pulposus (NP), a peripheral annulus fibrosus (AF), and a hyaline cartilage endplate (CEP) [3, 4]. Due to the nonvascular nature of the IVD, it can only obtain essential nutrients and excrete metabolites through the blood vessels of the CEP. When the CEP degenerates, the IVD is unable to undergo regular material exchange, resulting in IDD [5]. Therefore, CEP degeneration is considered to be one of the important causes of IDD [6]. Most treatments for IDD, including conservative treatments such as analgesics and surgical treatments such as spinal fusion, only address the clinical symptoms and have limited long-term effects [7]. Therefore, there is a need to find new therapeutic agents targeting pathogenesis to alleviate IDD and regenerate damaged IVD tissue by restoring tissue homeostasis. In recent years, traditional Chinese medicine (TCM) and other ethnic medicinal therapies have been increasingly used to prevent and treat various diseases, including IDD [8, 9].

Traditional Mongolian medicine (TMM) plays an indispensable role in benefiting the health of the Mongolian people, especially with its unique diagnosis and treatment of orthopaedic diseases, which has attracted increasing attention worldwide [10]. The Mongolian medicine Naru 3 pill has been used for at least 1000 years and consists of Caowu, Hezi, and Biba [11]. In the formula, Caowu can dispel “stickiness”, relieve “pain”, and dry “Huangshui”. Hezi can strengthen and detoxify the body. Biba can eliminate “Baganda and Heyi” and has the effects of warming meridians, dispersing cold and removing moisture. Naru 3 pill contains these three herbs and has the effects of eliminating “stickiness”, drying “Huangshui”, adjusting “Xieriwusu”, relieving pain, and regulating the balance of muscles and bones [12]. Naru 3 pill has a long history of clinical use in the treatment of acrodynia in wasted legs, rheumatism, and joint disease [11]. In addition, Naru 3 has also been used for the treatment of IDD [13]. However, the specific components and potential mechanisms by which Naru 3 pill protects against IDD remain unclear.

**Fig. 1:**
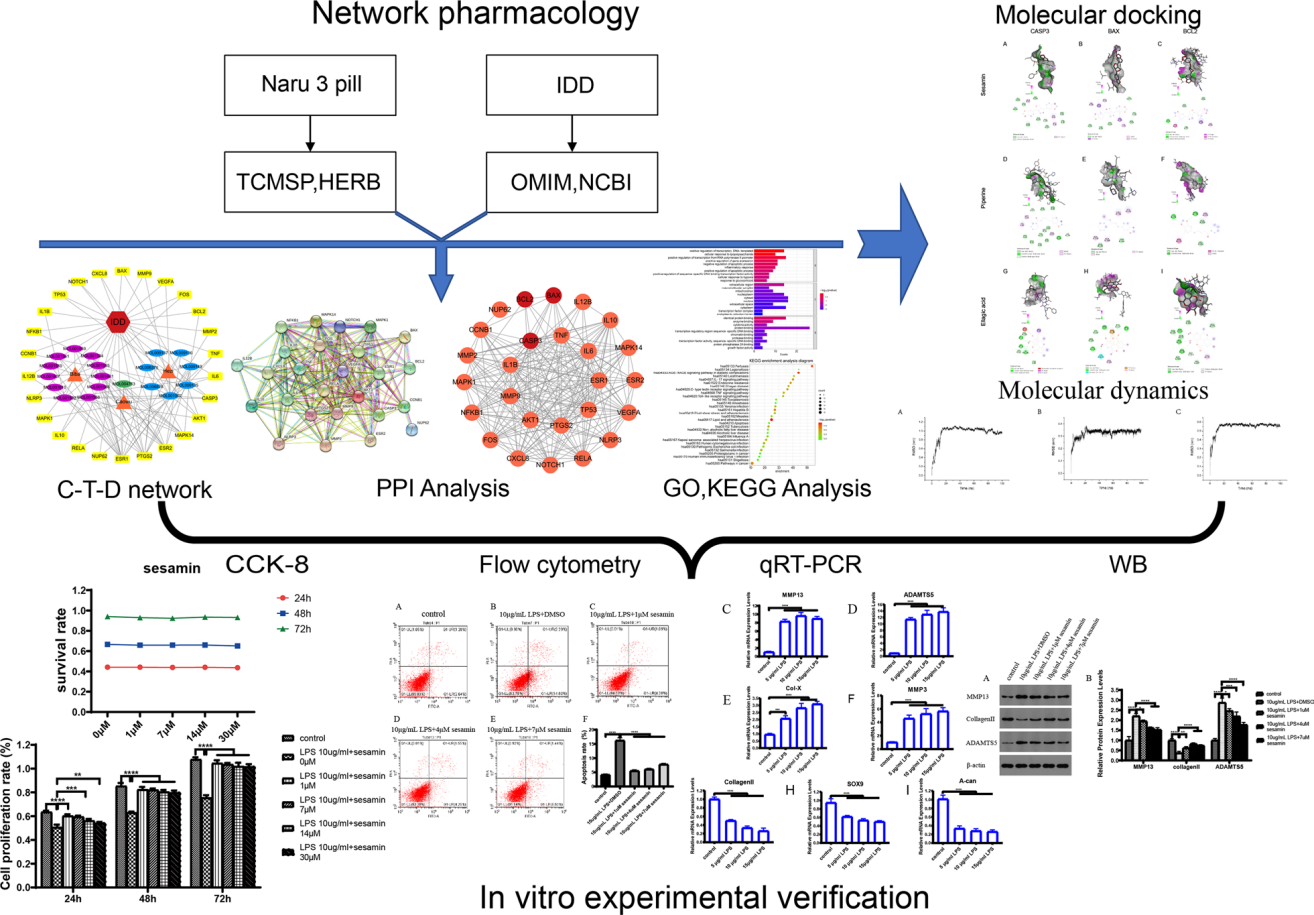
Flow chart showing the mechanism of Naru 3 pill in treating IDD. *IDD* Intervertebral disc degeneration, *TCSMP* Traditional Chinese Medicine Systematic Pharmacology Database and Analysis Platform, *OMIM* Online Mendelian Inheritance in Man, *NCBI* National Center for Biotechnology Information, *PPI* Protein‒protein interaction, *C-T-D* Component-target-disease, *GO* Gene ontology, *KEGG* Kyoto Encyclopedia of Genes and Genomes, *qRT‒PCR* Quantitative real-time polymerase chain reaction analysis, *WB* Western blot, *CCK-8* Cell counting kit 8

Network pharmacology is an emerging interdisciplinary subject that is a systematic approach to the holistic study of the mechanisms of drugs and diseases based on the construction of networks among drugs, components, targets, and diseases, and this strategy can provide new insights into drug treatment of diseases [14]. Molecular docking is a technique to assess the binding of compounds to target proteins and is often used together with network pharmacology to study pharmacological mechanisms [15]. Molecular dynamics simulation is a computer simulation of various ligand and receptor motions to assess stability and flexibility, etc. [16]. In this study, effective compounds and targets of Naru 3 pill for the treatment of IDD were screened by network pharmacology analysis, molecular docking and molecular dynamics simulations and were validated by in vitro experiments. The experimental procedure is shown in the flow chart (Fig. 1).

## Methods and materials

### Network pharmacology analysis

#### Screening active compounds and drug targets

Naru 3 pill is composed of Caowu, Hezi, and Biba. First, the active ingredients of Naru 3 pill were selected from the traditional Chinese medicine systems pharmacology database and analysis platform (TCMSP, https://old.tcmsp-e.com/tcmsp.php) based on oral bioavailability (OB) ≥ 30% and drug-likeness (DL) ≥ 0.18. Second, the targets of the active compounds in Naru 3 pill were obtained from the TCMSP database and the HERB database (http://herb.ac.cn/). Finally, a component-target (C-T) network of Naru 3 pill was constructed by Cytoscape 3.8.2 software.

#### Screening disease targets

Disease targets were obtained from the Online Mendelian Inheritance in Man (OMIM) (https://www.omim.org/) and the National Center for Biotechnology Information (NCBI) databases (https://www.ncbi.nlm.nih.gov) by entering the keyword “intervertebral disc degeneration”. Duplicates were eliminated to obtain the related targets of IDD.

#### Screening candidate targets

The potential targets of Naru 3 pill in the treatment of IDD were obtained by intersecting the targets of Naru 3 pill with IDD targets. The Venn diagram was constructed using the R package (version 4.2.0, Venn diagram package). Subsequently, a component-target-disease (C-T-D) network was drawn using Cytoscape 3.8.2 software. According to the degree of the nodes, the main compounds were screened.

#### Construction of the protein‒protein interaction network

The obtained targets were imported into the STRING database (https://www.string-db.org/) to obtain protein‒protein interaction (PPI) networks. The species was set as “Homo sapiens”, and the interaction score was set as 0.4. The generated TSV format files were then imported into Cytoscape 3.8.2 software for visualization and analysis.

#### Gene ontology enrichment analysis and Kyoto Encyclopedia of Genes and Genomes pathway enrichment analysis

Gene Ontology (GO) enrichment analysis and Kyoto Encyclopedia of Genes and Genomes (KEGG) pathway enrichment analysis were performed on the predicted targets using the DAVID database (https://david.ncifcrf.gov/). After the significance was set at *p* < 0.05, GO items (biological process (BP), cellular component (CC), molecular function (MF)) and KEGG signalling pathways were obtained. Then, online mapping was conducted using bioinformatics software (https://www.bioinformatics.com.cn).

#### Molecular docking

We downloaded the 3D structures of the receptor target proteins using the RCSB PDB database (https://www.rcsb.org/) [17]. The PDB format structures of BAX (PDB ID: 4BPM), BCL2 (PDB ID: 6OQB), CASP3 (PDB ID: 3KJF). The TCMSP database was then used to download the structures of the ligand complexes. The receptor proteins were dehydrated using PyMOL software, and then they were hydrogenated, and the charge was calculated using AutoDock software. The generated file was saved in PDBQT format. Finally, the receptor proteins were docked with the ligand compounds using AutoDock Vina.

#### Molecular dynamics simulation

Molecular dynamics simulations of the active ingredients and proteins identified by molecular docking were performed using Desmond software [18]. The small molecule topology files were constructed by ATB (http://atb.uq.edu.au/index.py). The topologies of BAX, BCL2, and CASP3 proteins were generated by the PDB2GMX tool, and the water model was an SPC/E solvent model with sodium ions to neutralize the system charge and make the system electrically neutral. Using periodic boundaries, firstly, the protein and small molecule ligand complexes were energy optimized for 1000 ps at 300 K. Secondly, for the optimized system, the NVT system equilibrium for 100 ps and NPT system equilibrium for 100 ps were performed, respectively, in which the system positions were all restricted, and finally the whole system was removed from position restriction at 300 K for The molecular dynamics simulation was performed at 300 K for 100 ns [19].

#### In vitro experimental verification

##### Cell culture

The ATDC5 cell line was obtained from the BeNa Culture Collection (Beijing, China) and was derived from the AT805 mouse embryonal carcinoma line. The cells were cultured in medium with 10% bovine serum (Gibco, NY, USA), and cell passage began after the primary cells reached 85% to 90% confluence. A single-cell suspension was prepared after digestion using a solution containing 0.25% trypsin–EDTA (Gibco, NY, USA), and the cells were passaged at a ratio of 1:3 in an incubator with constant saturated humidity (5% CO_2_, 37 °C) (Sanyo, Osaka, Japan) to expand the culture.

##### Cell processing

First, the cells were treated with 5 μg/mL, 10 μg/mL, and 15 μg/mL lipopolysaccharide (LPS) for 24 h to assess the effect of LPS (Sigma, MO, USA) on the degeneration of ATDC5 cells. The cells were then treated with 0 μg/mL, 10 μg/mL, 50 μg/mL, 100 μg/mL, or 150 μg/mL piperine (MCE, NJ, USA); 0 μM, 10 μM, 50 μM, 100 μM, or 150 μM ellagic acid (MCE, NJ, USA); or 0 μM, 1 μM, 7 μM, 14 μM, or 30 μM sesamin (MCE, NJ, USA) for 24 h after LPS stimulation. Next, normal ATDC5 cells were treated with the same concentrations of piperine, ellagic acid, and sesamin as described above to assess the toxicity of the drugs to normal cells. Finally, the effect of sesamin on LPS-induced cells was evaluated. The cells were divided into five groups: normal ATDC5 cells, LPS-treated ATDC5 cells + DMSO solvent, and LPS-treated ATDC5 cells + different concentrations of sesamin (1 μM, 4 μM, 7 μM).

##### Gene expression analysis by quantitative real-time polymerase chain reaction

The mRNA expression levels of MMP3, MMP13, collagen II, ADAMTS5, Col-X, SOX9, and A-can were measured. Total RNA was extracted from cells using TRIzol reagent (Ambion, Austin, TX, USA). Microspectrophotometry (Allsheng, Hangzhou, China) was used to measure the purity and concentration of the RNA samples. Reverse transcription of the RNA to cDNA was carried out using a HiScript II Reverse Transcriptase Kit (Vazyme, Nanjing, China). Quantitative real-time polymerase chain reaction was performed using SYBR Green PCR Master Mix (Vazyme, Nanjing, China), and the samples were predenatured at 95 °C for 10 min, followed by 40 cycles of denaturation at 95 °C for 15 s and annealing at 60 °C for 60 s. Finally, 10 μL of SYBR Green Master Mix, 0.4 μL of 50 × ROX Reference Dye II, 0.4 μL of 10 μM forward primer, 0.4 μL of 10 μM reverse primer, and 4 μL of cDNA were used, and the appropriate amount of water was added to reach a total reaction volume of 20 μL. β-Actin was used as an internal control, and the data were analysed using the 2^−ΔΔCt^ method. These experiments were repeated three times. The primers for qRT‒PCR in this study are listed in Additional file 1: Table S1.

##### Protein analysis (western blotting)

Proteins were isolated from cells with RIPA lysis solution (Beyotime, Shanghai, China). Protein concentrations were measured using a microplate reader (Thermo, Waltham, MA, USA). Total proteins were electrophoresed in 10% sodium dodecyl sulphate–polyacrylamide gels. After electrophoresis, the proteins were transferred onto 0.45 μM polyvinylidene fluoride membranes (PVDF, Millipore, Billerica, MA, USA) and then blocked with 5% skim milk for 2 h. Then, the membranes were incubated with the primary antibodies. The PVDF membranes were incubated with secondary antibodies (HRP-labelled antibodies, Boster, Wuhan, China) for 2 h at 37 °C. Protein bands were visualized using ECL substrate (Applygen, Beijing, China) and chemiluminescence. Band intensities were quantified using ImageJ software. All antibodies are shown in Additional file 2: Table S2.

##### CCK-8 assay

We measured the effect of active ingredients on degenerated cell viability and the toxicity in normal cells using a Cell Counting Kit-8 assay (CCK-8, MCE, NJ, USA). First, the cells were added to 96-well plates. Then, 10 μL of CCK-8 solution was added, and the culture plate was incubated for 3 h. Finally, the absorbance at 450 nm was measured using a microplate reader (Thermo, Waltham, MA, USA) to assess cell viability.

##### Apoptosis detection

Apoptosis was measured using a flow cytometer (Beckman Coulter, FL, USA) and Annexin V-FITC/PI Apoptosis Detection Kit (Vazyme, Nanjing, China). Briefly, the cells were placed into 6-well plates, digested with trypsin, and then washed twice with phosphate-buffered saline (PBS). Subsequently, the cells were collected and resuspended in 500 μL of binding buffer, followed by incubation in 5 μL of Annexin V-FITC and 5 μL of PI for 15 min under a light. Finally, the cells were observed and detected by flow cytometry (Beckman Coulter, FL, USA).

##### EdU analysis

Cell proliferation was detected by flow cytometry with the EdU-647 Cell Proliferation Assay Kit (Beyotime, Shanghai, China). First, the cells were added to 6-well plates, digested with trypsin, and washed twice with PBS. Afterwards, the cells were collected and resuspended in 500 μL of binding buffer, and then 10 μM EdU working solution and 0.5 mL of the click reaction solution were added and incubated for 30 min at room temperature in the dark. Finally, the cells were observed and detected by flow cytometry (Beckman Coulter, FL, USA).

### Statistical analysis

The data are presented using the standard deviation (SD) and the standard error of the mean (SEM). Since the data were normally distributed, statistical analysis was performed using one-way analysis of variance (ANOVA). GraphPad Prism 9 software was used for analysis and graphing. A *p* value < 0.05 indicated a significant difference.

## Results

### Network pharmacology analysis

#### Active components of Naru 3 pill and component-target network analysis

**Fig. 2:**
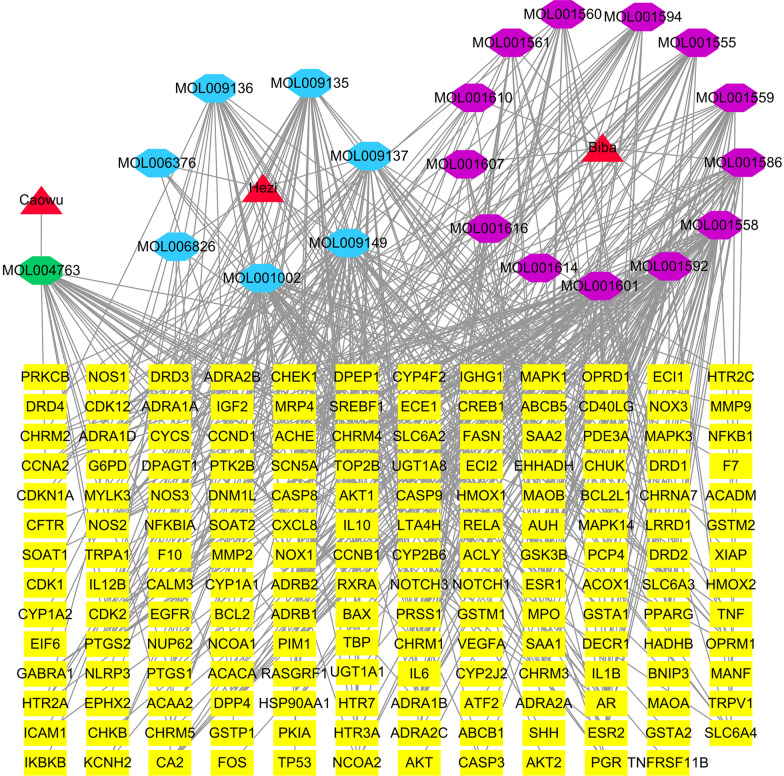
C-T network of Naru 3 pill. The yellow rectangles represent the 167 corresponding targets of the compounds. The green, blue, and purple hexagons are compounds in Caowu, Hezi, and Biba, respectively. The red triangle indicates Caowu, Hezi, and Biba

The components of Naru 3 pill include three herbs: Caowu, Biba, and Hezi. In this study, 21 bioactive compounds were screened from the TCMSP database using the following parameters: OB ≥ 30% and DL ≥ 0.18. Among them, 1 active compound was in Caowu, 13 were in Biba, and 7 were in Hezi (Table 1). A total of 468 potential targets were obtained from the TCMSP and HERB databases, and 167 targets were retained after removing duplicates. We then used Cytoscape 3.8.2 to create a component-target (C-T) network (Fig. 2). The yellow rectangles represent 167 targets. The green, blue, and purple hexagons represent the compounds of Caowu, Hezi, and Biba, respectively. The red triangles represent Caowu, Hezi, and Biba.

**Table 1:** Active ingredient in Naru 3 Pill

Herb	Mol ID	Molecule Name	MW	OB (%)	DL
Caowu	MOL004763	Izoteolin	327.41	39.53	0.51
Hezi	MOL001002	ellagic acid	302.2	43.06	0.43
Hezi	MOL006376	7-Dehydrosigmasterol	414.79	37.42	0.75
Hezi	MOL006826	chebulic acid	356.26	72	0.32
Hezi	MOL009135	ellipticine	246.33	30.82	0.28
Hezi	MOL009136	Peraksine	310.43	82.58	0.78
Hezi	MOL009137	(R)-(6-methoxy-4-quinolyl)-[(2R,4R,5S)-5-vinylquinuclidin-2-yl]methanol	324.46	55.88	0.4
Hezi	MOL009149	Cheilanthifoline	325.39	46.51	0.72
Biba	MOL001559	piperlonguminine	273.36	30.71	0.18
Biba	MOL001607	ZINC03982454	414.79	36.91	0.76
Biba	MOL001601	1,2,5,6-tetrahydrotanshinone	280.34	38.75	0.36
Biba	MOL001592	piperine	285.37	42.52	0.23
Biba	MOL001614	(E,E,E)-11-(1,3-Benzodioxol-5-yl)-N-(2-methylpropyl)-2,4,10-undecatrienenamide	353.5	42.72	0.43
Biba	MOL001610	sylvatine	383.58	44	0.51
Biba	MOL001561	dehydropipernonaline	339.47	47.73	0.41
Biba	MOL001616	1-[1-oxo-9(3, 4-methylenedioxyphenyl)-2E,8E-nonadienyl] pyrrolidine	327.46	49.43	0.36
Biba	MOL001560	pipernonaline	341.49	51.32	0.41
Biba	MOL001555	ZINC03996196	386.48	52.35	0.62
Biba	MOL001558	sesamin	354.38	56.55	0.83
Biba	MOL001586	N-(2,5-dimethoxyphenyl)-4-methoxybenzamide	287.34	60.7	0.18
Biba	MOL001594	Pisatin	314.31	88.05	0.64

*MW* Molecular weight, *OB* Oral bioavailability, *DL* Drug-likeness

#### Component-target-disease network analysis

**Fig. 3:**
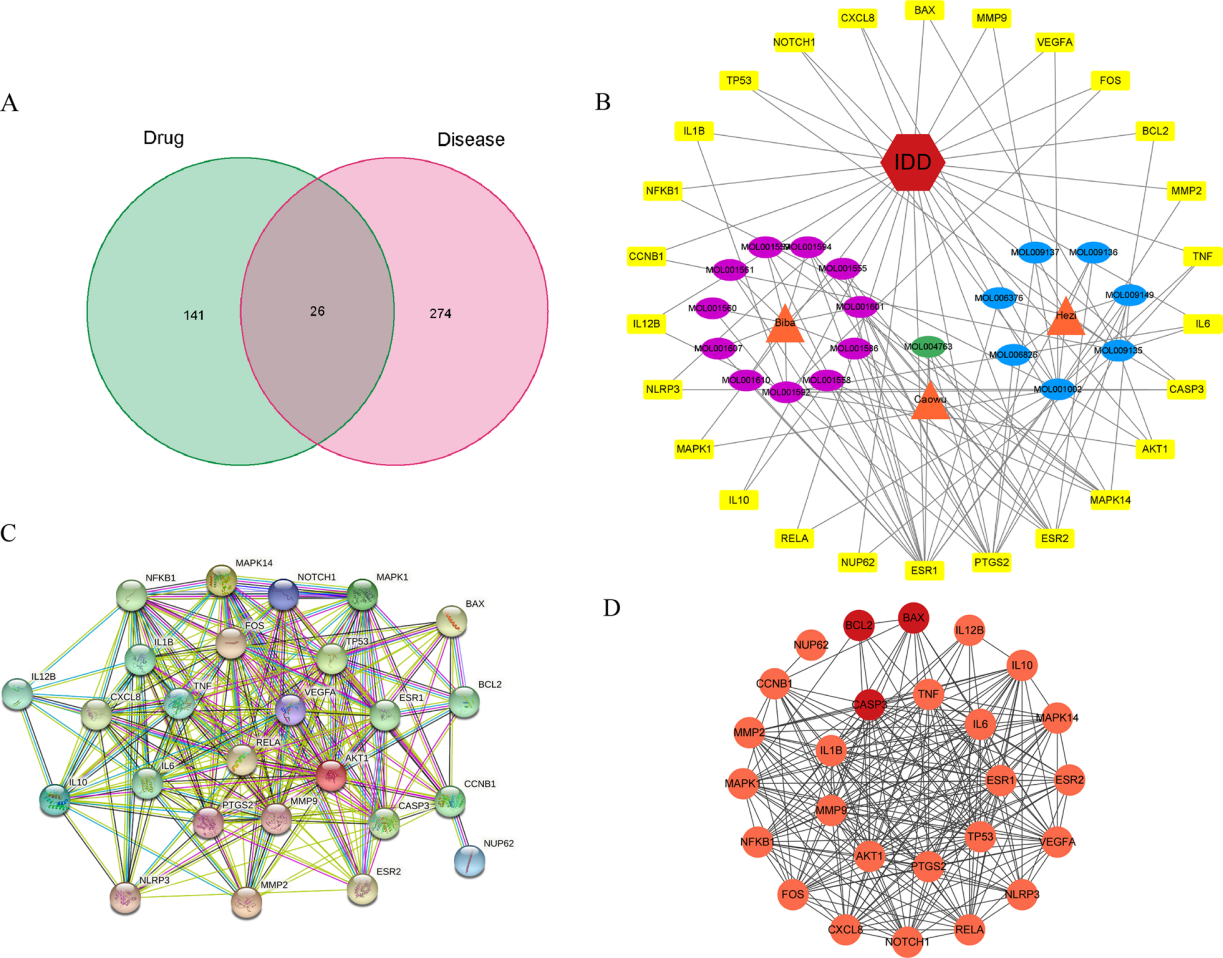
Analysis of key targets by which Naru 3 pill treats lumbar degeneration. (**A**) After obtaining 167 targets of Naru 3 pill and 300 targets of IDD, we overlapped the two sets of targets and obtained 26 mutual targets. (**B**) Component-target-disease network. (**C**) The PPI network of the 26 overlapping targets was analysed and constructed by STRING. (**D**) A simplified PPI Network was constructed with Cytoscape

IDD targets were retrieved from the OMIM and NCBI databases, and 300 targets were obtained after removing duplicates. We intersected the 167 targets of Naru 3 pill with the 300 targets of IDD and obtained 26 intersected targets, which were considered potential targets of Naru 3 pill for the treatment of IDD and are presented using a Venn diagram (Fig. 3A). Then, a component-target-disease network was constructed using Cytoscape 3.8.2 (Fig. 3B). The yellow rectangles represent the 26 corresponding targets. The green, blue and purple ovals represent the compounds of Caowu, Hezi, and Biba. The orange triangles represent Caowu, Hezi, and Biba, and the red hexagon represents IDD. In the network, higher degrees indicated more critical nodes. The compounds ranked at the top of the degree list were sesamin, piperine, ellagic acid, and ellipticine, which might play crucial roles in the effect of Naru 3 pill on the treatment of IDD. Ellipticine was excluded because it was rarely reported in the literature related to IDD.

#### PPI network analysis

The PPI network was constructed by entering the 26 targets into STRING 11.5 and setting the confidence level to 0.4 (Fig. 3C). The network was then visualized in Cytoscape 3.8.2. In the PPI network, targets with a high degree played a vital role in the central correlation. We screened the top-ranked targets, including AKT1, TNF, CASP3, PTGS2, ESR1, TP53, MMP9, IL1B, and IL6 (Fig. 3D).

#### GO analysis

**Fig. 4:**
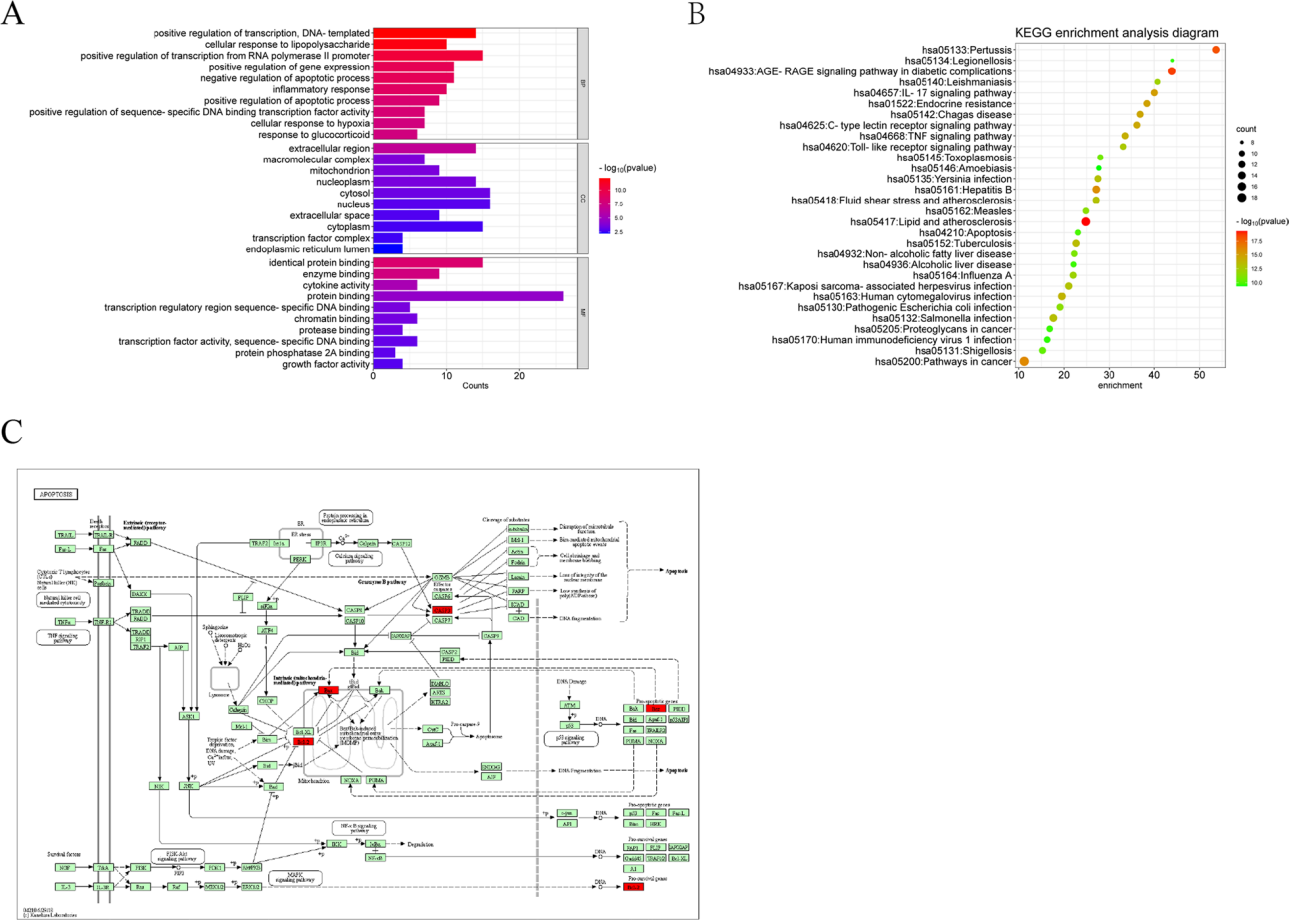
GO enrichment and KEGG pathway enrichment analysis of the 26 potential therapeutic targets. (**A**) GO enrichment analysis: The top 10 BP, CC, and MF terms ranked according to the *p* value; the larger the *p* value, the darker purple the colour. (**B**) Bubble diagram showing KEGG enrichment of potential targets of Naru 3 pill for the treatment of IDD. The larger the number of enriched targets, the larger the dots; the larger the *p* value, the darker green the dot colour. (**C**) Schematic diagram of apoptotic pathways marked in red as key targets

GO analysis was performed on 26 targets based on the DAVID database. A total of 313 GO terms were obtained (*p* < 0.05), including 263 BP, 35 MF, and 15 CC terms. The top-ranked BP terms were related to the regulation of apoptotic processes, inflammatory responses, cellular responses to lipopolysaccharides, senescence, the positive regulation of transcription, and DNA templates, while the top-ranked MF terms were related to protein binding, enzyme binding, sequence-specific DNA binding in transcriptional regulatory regions, and transcription factor activity. CC enrichment was mainly related to extracellular regions, mitochondria, and macromolecular complexes. The top 10 BP, CC, and MF terms were ranked based on *p* values and are shown in Fig. 4A.

#### KEGG pathway enrichment analysis

A total of 123 significantly enriched signalling pathways were obtained by KEGG enrichment analysis (*p* < 0.05), and the top 5 pathways were lipids and atherosclerosis, the AGE-RAGE signalling pathway in diabetic complications, pertussis, the cancer pathway, and hepatitis B. The top 30 pathways are visually represented in the bubble plots in Fig. 4B. The results indicated that apoptotic pathways were closely related to IDD. After integrating target prediction, pathways, and functional enrichment, CASP3, BAX, and BCL2 were considered highly associated with apoptosis and enriched in apoptotic pathways (Fig. 4C). Our study suggested that these three targets could be the main targets of Naru 3 pill in the treatment of IDD, which might be achieved by regulating apoptosis. The expression of these three key targets was later examined in vitro.

#### Molecular docking

**Fig. 5:**
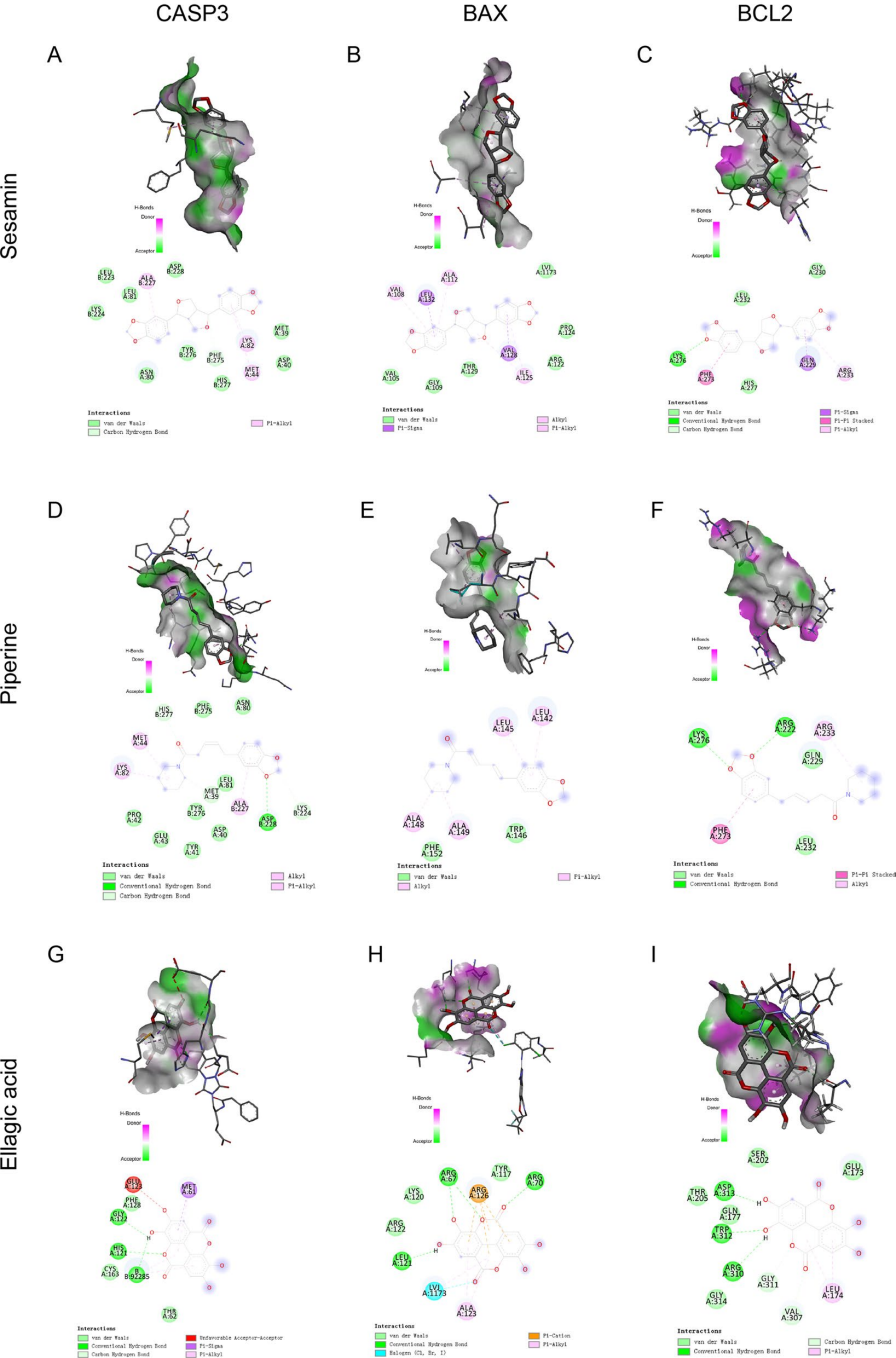
Molecular docking diagram of a small molecule compound with a target protein. (**A**–**C**) The simulated docking diagram of sesamin with CASP3, BAX and BCL2 is shown. (**D**–**F**) The simulated docking diagram of piperine with CASP3, BAX and BCL2 is shown. (**G**–**I**) The simulated docking diagram of ellagic acid with CASP3, BAX and BCL2 is shown

We molecularly docked three small molecules with three apoptotic targets (Fig. 5). It is generally believed that the smaller the ligand‒receptor binding energy is, the better the mutual binding effect. The results showed that the docking affinities of sesamin, piperine, and ellagic acid with the apoptotic proteins CASP3, BAX, and BCL2 were all less than -5 kJ/mol, indicating that these factors all bound well to apoptotic targets, among which sesamin bound the best (Table 2).

**Table 2:** Binding energy of the compound with the target protein

Compound	CASP3 (kcal/mol)	BAX (kcal/mol)	BCL2 (kcal/mol)
sesamin	− 7.2	− 7.2	− 7.2
piperine	− 6.1	− 6.8	− 6.2
ellagic acid	− 6.3	− 5.9	− 6.5

*CASP3* Cysteinyl aspartate specific proteinase-3, *BAX* BCL2-associated X protein, *BCL-2* B-cell lymphoma-2

#### Molecular dynamics simulation

**Fig. 6:**
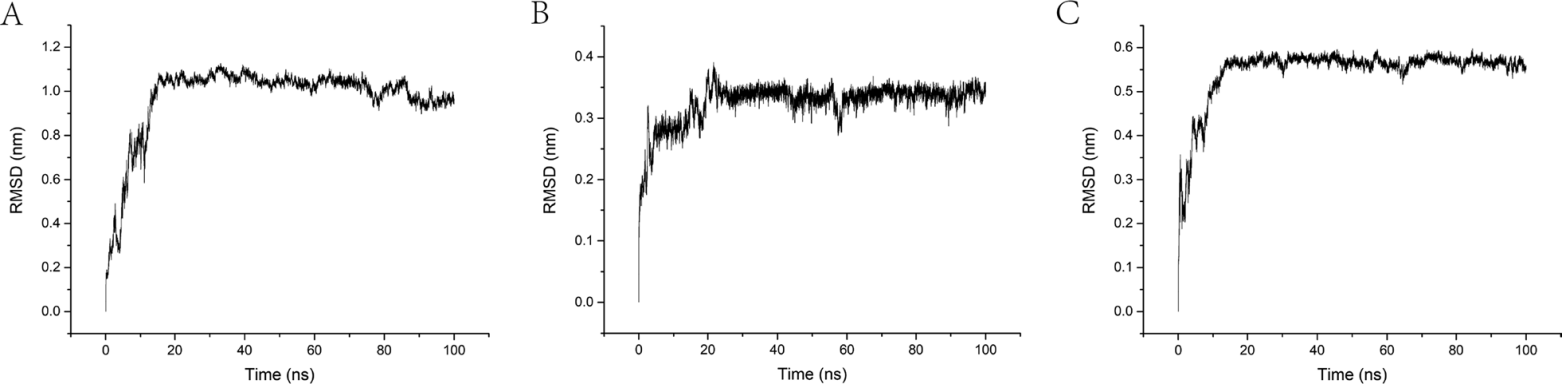
RMSD plot during molecular dynamics simulations. (**A**) The RMSD of BAX-sesamin. (**B**) The RMSD of BCL2-sesamin. (**C**) The RMSD of CASP3-sesamin

The root mean square deviation (RMSD) is a good measure of the conformational stability of protein and ligands and is a measure of the extent of deviation in the position of atoms from the starting position. A lower deviation indicates a better conformational stability. The changes in RMSD values of the complexes were analysed. The RMSD value of the BAX-sesamin system fluctuated around 0.1 nm after 20 ns, which proved that the BAX-sesamin conformation had basically reached stability (Fig. 6A). The RMSD value of the BCL2-sesamin system fluctuated around 0.33 nm after 60 ns and gradually reached equilibrium (Fig. 6B). The RMSD value of CASP3-sesamin system was basically unchanged after 20 ns and fluctuated in the range of 0.56 nm and basically reached the equilibrium state (Fig. 6C). The above results show that the RMSD values of these three systems basically converge and the systems are stable and reasonable during the MD simulation.

#### In vitro experimental verification

##### LPS induces ATDC5 cell degeneration

**Fig. 7:**
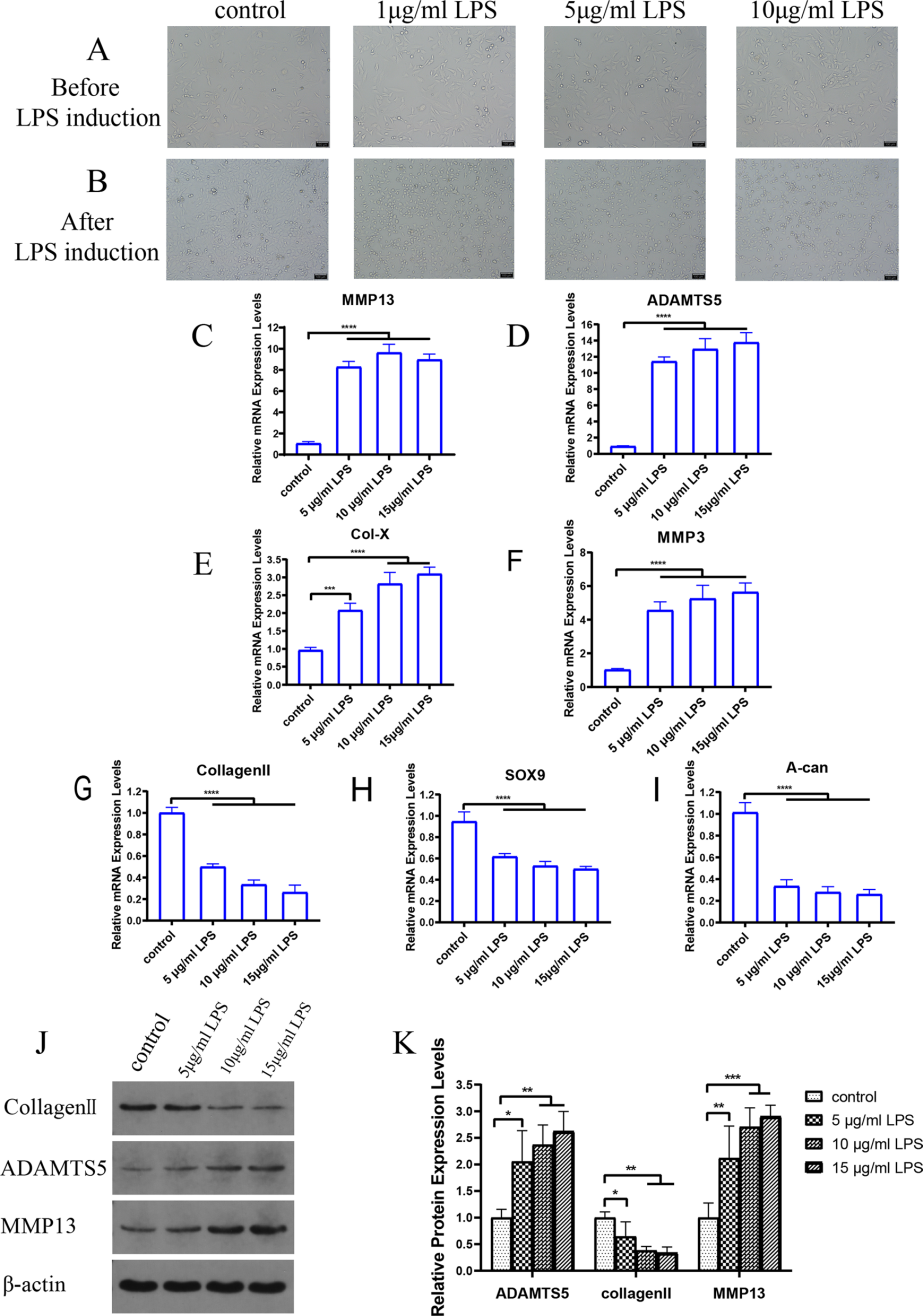
LPS induces ATDC5 cell degeneration. (**A**,**B**) The morphological changes in ATDC5 cells before and after induction with different concentrations of LPS were observed by microscopy. (**C**–**I**) Effect of LPS on the mRNA expression of MMP3, MMP13, collagen II, ADAMTS5, Col-X, SOX9, and A-can in ATDC5 cells. ****p < 0.0001 compared with the blank control group. (**J**,**K**) Effect of LPS on the protein expression of MMP13, collagen II, and ADAMTS5 in ATDC5 cells. *p < 0.05; **p < 0.01; ***p < 0.001 compared with the blank control group

ATDC5 cells were treated with different concentrations of LPS (0, 5, 10, and 15 μg/mL) for 24 h. The results showed that different concentrations of LPS could induce different degrees of cell degeneration (Fig. 7A,B). The PCR results showed that LPS dramatically increased the gene expression levels of MMP13, MMP3, ADAMTS5, and Col-X in ATDC5 cells and reduced the gene expression levels of collagen II, A-can, and SOX9, and the difference was more significant with increasing LPS concentrations (Fig. 7C–I). The WB results showed that the protein expression levels of MMP13 and ADAMTS5 were increased, while the expression levels of collagen II were decreased, and the most significant effect was observed at LPS concentrations of 10 μg/mL and 15 μg/mL (Fig. 7J,K). These findings revealed that LPS could induce the degeneration of ATDC5 cells, and we chose 10 μg/mL LPS to induce ATDC5 cell degeneration.

##### Effects of drug monomers on ATDC5 cell proliferation in degeneration models

**Fig. 8:**
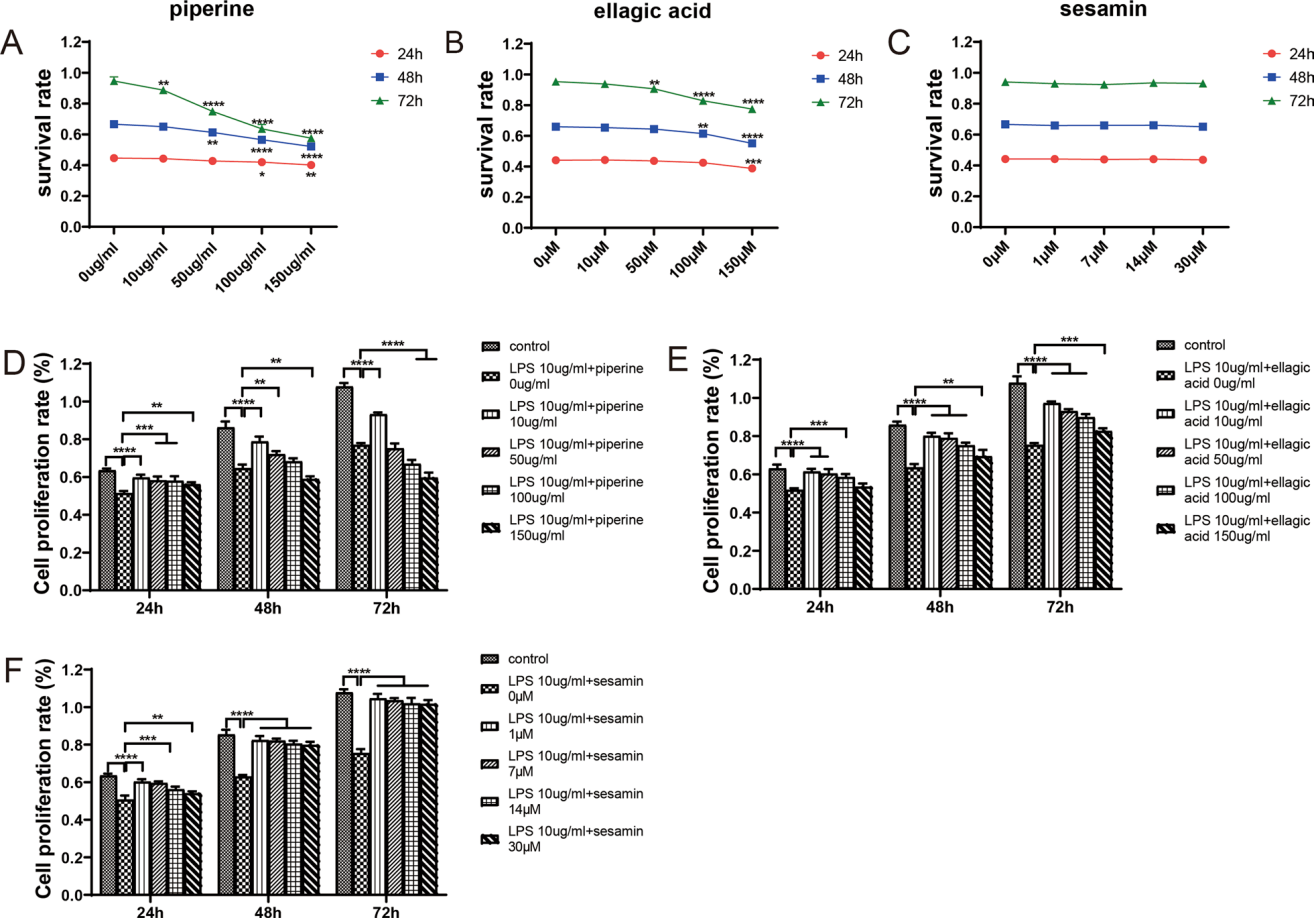
Effects of drug monomers on ATDC5 cell proliferation in degeneration models. (**A**–**C**) Toxicity of the active ingredients on normal cells (24 h, 48 h, and 72 h). *p < 0.05; **p < 0.01; ***p < 0.001; ****p < 0.0001 compared with the 0 dose group. (**D**–**F**) Effects of pharmaceutically active ingredients on the proliferation of LPS-induced ATDC5 cells. **p < 0.01; ***p < 0.001; ****p < 0.0001 compared with the 0 dose group

The network pharmacology results demonstrated that sesamin, piperine, and ellagic acid might be the main active compounds of Naru 3 pill in the treatment of IDD. We assessed the effects of sesamin, piperine, and ellagic acid on the cytotoxicity of ATDC5 cells using the CCK-8 assay and found that none of the three monomers had an effect on cells at 24 h, whereas piperine and ellagic acid were more toxic at 72 h. In contrast, sesamin had no effect on cells during and of the tested time periods (Fig. 8A–C).

We then carried out a CCK-8 assay to evaluate the influence of sesamin, piperine, and ellagic acid on LPS-induced ATDC5 cell proliferation. The CCK-8 results showed that LPS inhibited the proliferation of ATDC5 cells. As shown in Fig. 8D, 10 μg/mL piperine significantly promoted cell proliferation in vitro during all three time periods, while the effect of 50 μg/mL, 100 μg/mL and 150 μg/mL piperine on promoting proliferation gradually diminished and even showed the opposite effect with time. At 72 h, 100 μg/mL piperine inhibited cell proliferation, and 150 μg/mL piperine inhibited cell proliferation at both 48 h and 72 h. Similarly, 10 μM, 50 μM, 100 μM, and 150 μM ellagic acid significantly promoted cell proliferation, and this effect decreased with increasing concentrations; 10 μM ellagic acid had the most pronounced effect, while 150 μM ellagic acid did not promote cell proliferation at 24 h (Fig. 8E). Furthermore, 1 μM, 7 μM, 14 μM, and 30 μM sesamin significantly promoted cell proliferation compared with the other drug monomers, and there was no significant difference in the effect at different concentrations with prolonged treatment times. Thus, sesamin promoted proliferation most effectively (Fig. 8F). Therefore, we conducted subsequent experiments with sesamin.

##### Effects of sesamin on the expression of degeneration-related proteins in ATDC5 cells

**Fig. 9:**
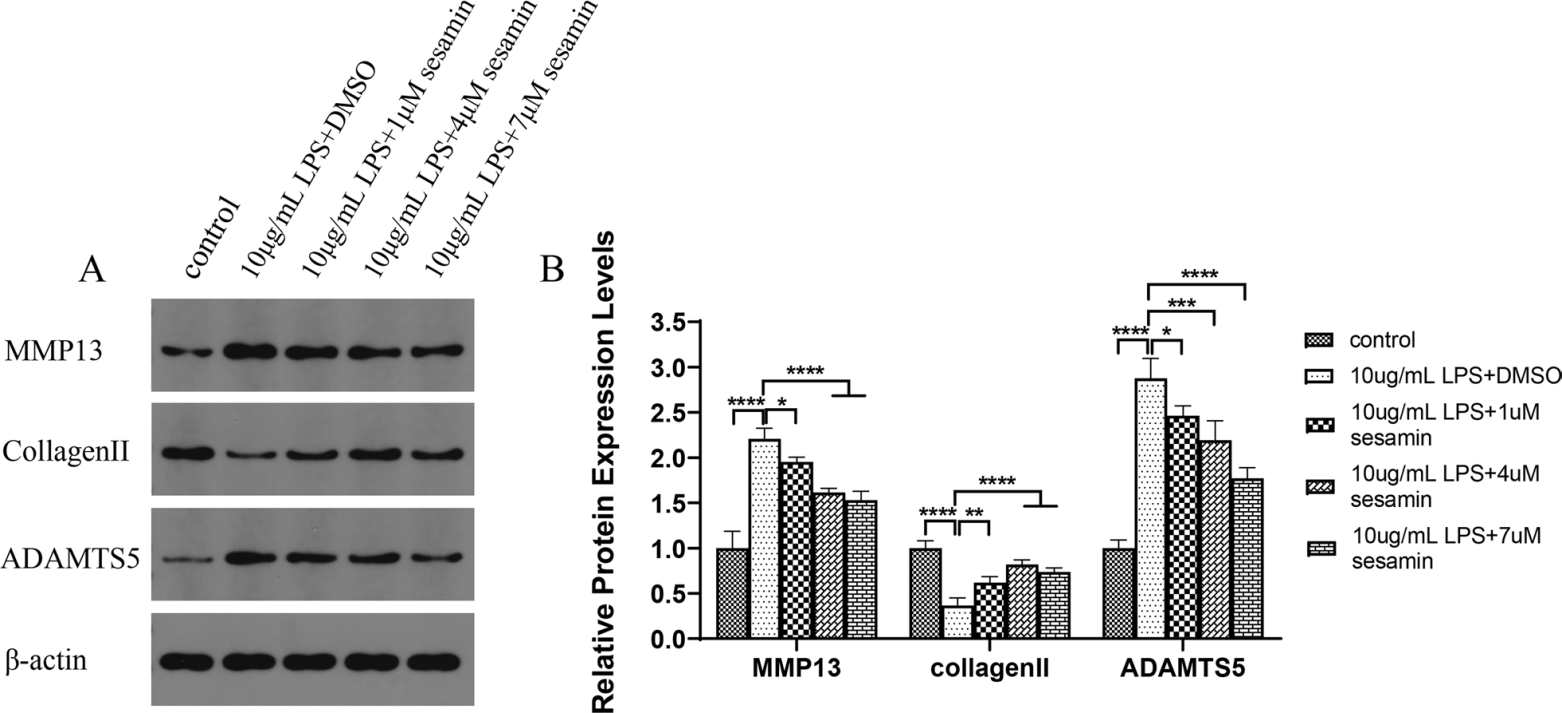
Sesamin treatment inhibits LPS-induced degeneration in ATDC5 cells. (**A**,**B**) The protein levels of Bax, Bcl-2, and caspase9 were assessed by western blotting. *p < 0.05; **p < 0.01; ***p < 0.001; ****p < 0.0001 compared with the 10 μg/mL LPS + DMSO group

The effect of sesamin on LPS-induced extracellular matrix (ECM) degradation in ATDC5 cells was assessed by WB analysis. As shown in Fig. 9A,B, the expression of MMP13 and ADAMTS5 was elevated after LPS stimulation, while the expression of collagen II was decreased. Compared with that in the LPS + DMSO group, 1 μM, 4 μM and 7 μM sesamin inhibited the expression of MMP13 and ADAMTS5 and enhanced collagen II expression in LPS-stimulated ATDC5 chondrocytes, and 7 μM sesamin regulated the expression of MMP13, ADAMTS5 and collagen II most significantly.

##### Effects of sesamin on ATDC5 cell apoptosis and the expression of key apoptotic targets

**Fig. 10:**
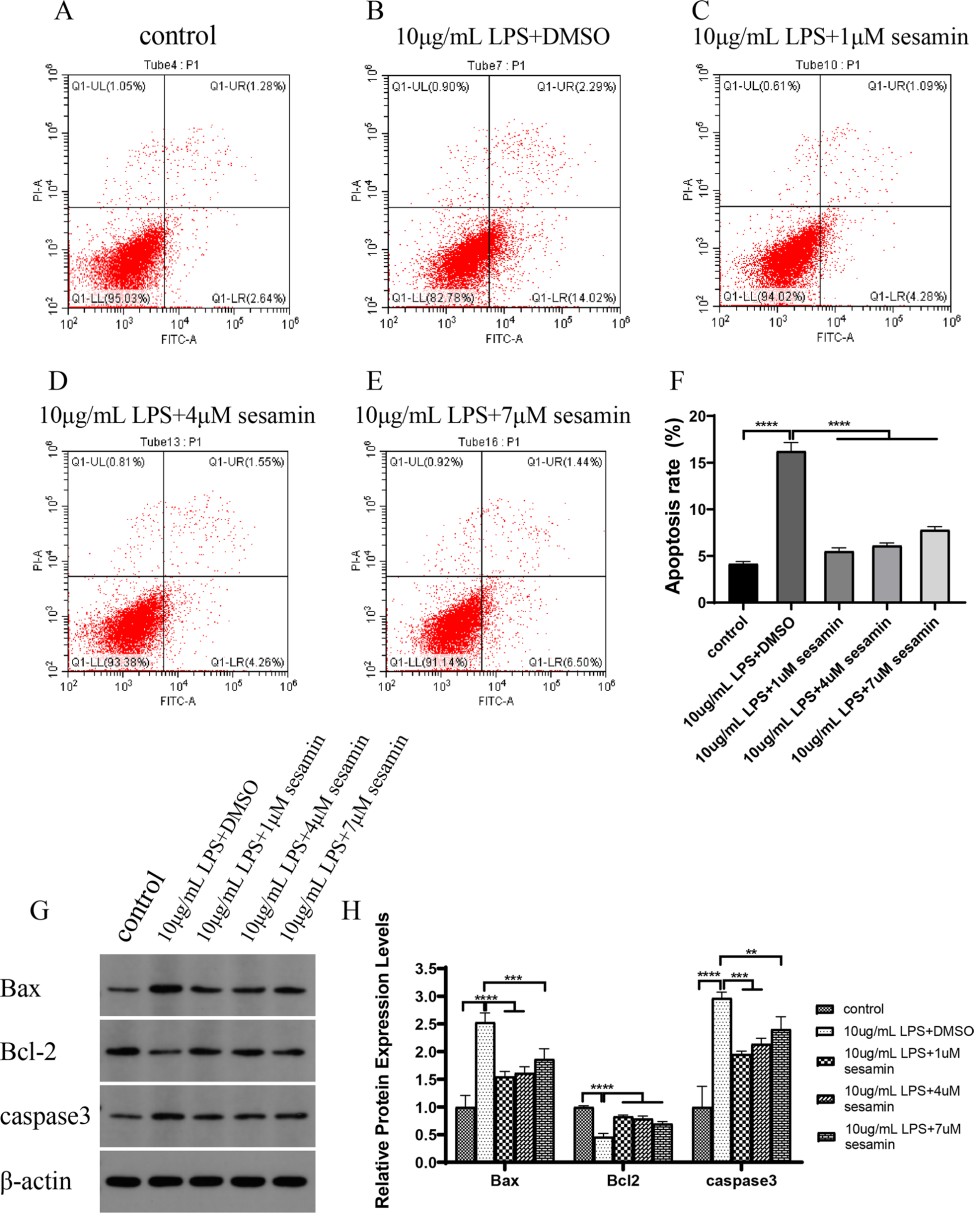
Sesamin treatment delayed LPS-induced apoptosis in ATDC5 cells. (**A**–**F**) Cell apoptosis was analysed by flow cytometry. ****p < 0.0001 compared with the 10 μg/mL LPS + DMSO group. (**G**,**H**) The protein levels of Bax, Bcl-2, and caspase9 were assessed by western blotting. **p < 0.01; ***p < 0.001; ****p < 0.0001 compared with the 10 μg/mL LPS + DMSO group

Flow cytometry showed that LPS significantly promoted ATDC5 cell apoptosis compared with that in the control group. However, sesamin dramatically reversed the effect of LPS on ATDC5 cell apoptosis and reduced the level of apoptosis (Fig. 10A–F). In addition, sesamin decreased the protein expression levels of the proapoptotic factors BAX and CASP3 in LPS-stimulated ATDC5 cells compared to those in the LPS group but increased the protein expression levels of Bcl-2 (Fig. 10G,H). These experiments fully validated the network pharmacology findings and confirmed the expression profile of the predicted targets and the regulation of apoptosis.

##### Effects of sesamin on ATDC5 cell proliferation as detected by flow cytometry

**Fig. 11:**
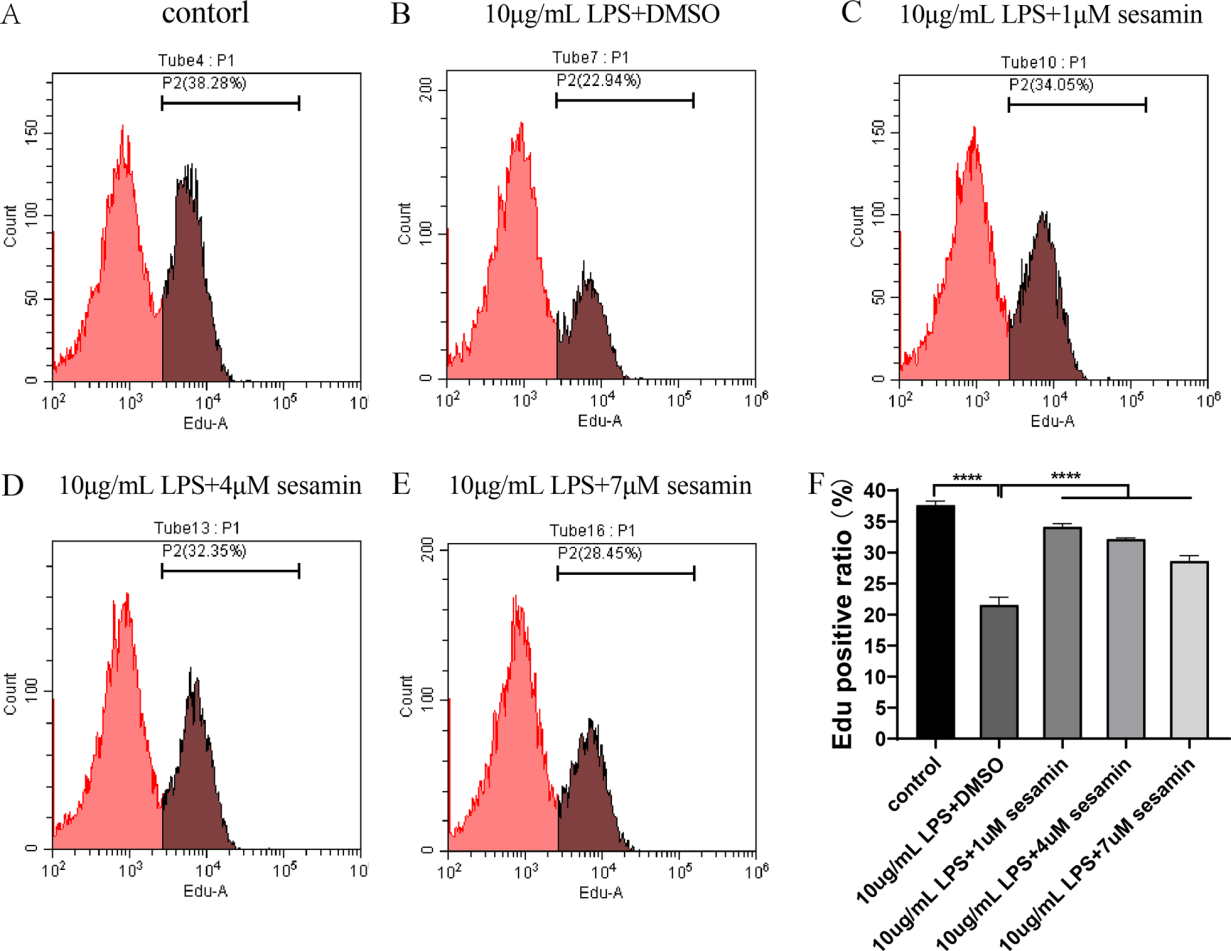
Sesamin treatment promoted LPS-induced proliferation in ATDC5 cells and decreased the rate of EdU-positive cells. (**A**–**F**) Cell proliferation and the rate of EdU-positive cells were analysed by flow cytometry. ****p < 0.0001 compared with the 10 µg/mL LPS + DMSO group

Compared with untreated cells, LPS-treated cells showed a significant increase in the EdU-positive rate and reduced DNA replication, while the sesamin-treated group showed a significant decrease in the EdU-positive rate and increased DNA replication. Our results suggested that sesamin increased the proliferation of LPS-induced ATDC5 cells (Fig. 11A–F).

## Discussion

LBP is the number one cause of multiyear disability, and IDD is an essential reason for its occurrence. The mechanisms of IDD are complex, and CEP degeneration is thought to be closely related to the development of IDD [3, 20]. Currently, most treatments for IDD are limited to symptomatic treatments, and there is still no practical way to reverse or delay the progression of this disease [21]. Naru 3 pill has a long history of treating musculoskeletal disorders [11]. However, the specific mechanism of Naru 3 pill is not clear, which limits its clinical application. This study comprehensively and systematically explored and explained the mechanism of Naru 3 pill in the treatment of IDD through network pharmacology analysis. First, a component-target-disease network was constructed, and three active compounds with many nodes were selected. Second, a Venn diagram of the intersections of drug targets and disease targets was constructed to obtain 26 targets, and the PPI network was further built and visualized. Finally, 313 GO items and 123 related signalling pathways were obtained through GO and KEGG enrichment analyses, and the binding of small molecule compounds to target proteins was conducted using molecular docking methods. In addition, the mechanism of the active compounds of Naru 3 pill in the treatment of IDD was explored by in vitro validation experiments. Therefore, through network pharmacology analysis, molecular docking, Molecular dynamics simulation and cell validation experiments, the molecular mechanism of Naru 3 pill in the treatment of IDD was revealed.

The results of the network pharmacology study showed that piperine, ellagic acid, and sesamin might be the active components in Naru 3 pill that played therapeutic roles in IDD. The results of molecular docking also revealed that these three compounds had excellent binding effects with the target proteins. Piperine has an anti-inflammatory effect and can inhibit the expression levels of inflammatory factors (iNOS, COX-2) and degenerative factors (MMP3, MMP13) in IL-1β-stimulated osteoarthritic chondrocytes, thereby treating osteoarthritis. The anti-inflammatory effect of piperine may be achieved by inhibiting the activity of NF-κB [22]. In a clinical study, it was found that after the application of a herbal preparation containing piperine, knee osteoarthritis in patients was improved [23]. Pepper salt may also treat sciatica by modulating inflammation [24]. Ellagic acid is a polyphenol that is present in a variety of natural substances. It achieves its anti-inflammatory effect by inhibiting the NF-κB pathway and inhibiting the IL-1β-induced inflammatory response in human chondrocytes. In addition, ELLAGIC acid also decreases MMP13 and ADAMTS5 and increases collagen II and aggregated proteoglycans. Ultimately, the progression of osteoarthritis can be delayed [25]. Another study showed that ellagic acid relieves arthritis through its anti-inflammatory, antiangiogenic, and antiapoptotic effects [26]. These findings demonstrate the potential role of ellagic acid in slowing the progression of osteoarthritis. Sesamin, a lipid-soluble lignan, can inhibit IL-1β signalling in chondrocytes to achieve its anti-inflammatory effect and inhibit the degradation of collagen II and proteoglycans, which can treat osteoarthritis [27]. Sesamin has been proven to inhibit LPS-induced IVD inflammation and ECM catabolism in rats. Intra-IVD injection of sesamin in a rat model of IDD inhibits the reduction in NP signals on MRI T2-weighted images and the upregulation of MMP3 and ADAMTS5 mRNA expression. This finding suggests that intra-IVD injection of sesamin can effectively maintain the typical morphology of the IVD and inhibit the pathological changes of IDD, demonstrating the protective effect of sesamin on IDD [28]. In the present study, the CCK-8 assay showed that piperine, ellagic acid and sesamin induced proliferation in LPS-stimulated ATDC5 cells. However, high concentrations of piperine inhibited proliferation, and the effect of ellagic acid on promoting proliferation decreased with increasing concentrations. In contrast, the proliferative effect of sesamin was highly significant. In addition, ellagic acid and piperine had different degrees of inhibitory effects on the growth of normal ATDC5 cells at high concentrations over time, while sesamin had no inhibitory effect on the growth of normal ATDC5 cells and markedly promoted the proliferation of degenerated cells. Moreover, the molecular docking results showed that sesamin bound best to the three target proteins. The results of molecular dynamics simulations indicated that the three systems BAX-sesamin, BCL2-sesamin and CASP3-sesamin were stable and reasonable during the simulation. Therefore, our findings indicated that sesamin might play an important role in the effects of Naru 3 pill and further explored its mechanism of action.

We performed a PPI visualization network analysis and found that CASP3, BAX, and BCL2 were most closely related to the apoptotic pathway and were identified as essential target proteins. The KEGG results suggested that the apoptotic pathway played a vital role in Naru 3 pill-mediated treatment of IDD, and this pathway was closely related to IDD [7]. The results of GO analysis demonstrated that biological processes regulating apoptosis played crucial roles. Apoptosis is a programmed mode of death and an important mechanism for maintaining tissue structure and function [29]. Apoptotic pathways include exogenous death receptor pathways, endogenous mitochondrial pathways, and ERS pathways. Apoptosis plays an indispensable role in the occurrence and development of IDD, and the regulation of apoptosis is an attractive therapeutic strategy for IDD [30]. Cysteinyl aspartate specific proteinase-3 (Caspase-3, CASP3) is an essential protein in apoptosis that is activated by both endogenous and exogenous death pathways [31]. CASP3 is the terminal executor protease in apoptosis that initiates the breakdown of cellular components by cleaving structural proteins, disrupting nuclear membranes, and damaging genomic DNA through the activation of cysteine protease-activated deoxyribonucleases. In addition, cells have intrinsic mechanisms for controlling cysteinase activity because low levels of CASP3 activity are critical for many cell types in developmental processes. Therefore, various strategies have been developed to regulate CASP3 activity to allow this developmental process to proceed without triggering cell death [32]. It has been demonstrated that short-term inhibition of CASP3 can treat injury-induced IDD but accelerates the progression of senescence-induced IDD [33]. B-cell lymphoma-2 (BCL2) has been identified in acute lymphoblastic leukaemia and has later been shown to inhibit apoptosis, while BCL2-associated X (BAX) is a well-known proapoptotic factor [34]. These factors all belong to the BCL2 family. The BCL2 homology (BH) structural domain is characteristic of this family. The interactions within the BCL2 family are crucial for the regulation of apoptosis [35]. Mitochondrial membrane permeabilization is an essential link in the mechanism of apoptosis. BAX can promote apoptosis by inducing mitochondrial membrane permeabilization [36]. Therefore, regulating the balance between BCL2 family proteins is critical for shaping cell life and death [37]. Sesamin is used to inhibit cancers by inducing apoptosis in liver, breast, and lung cancer cells [38]. Conversely, sesamin may also treat femoral head necrosis by inhibiting apoptosis in osteoblasts [39]. Our study showed that sesamin inhibited apoptosis in degenerated ATDC5 cells and the protein expression of BAX and CASP3 and promoted the protein expression of Bcl-2. In this study, we experimentally determined the expression levels of apoptotic factors and related apoptotic protein targets. The results of the network pharmacology analysis were validated. This finding fully illustrated the apoptosis-delaying effect of sesamin, which is the main component of Naru 3 pill. Our study demonstrated the potential role of Naru 3 pill in the treatment of IDD, which provides a new idea for the treatment of IDD.

The structure and function of the ECM are crucial for IVD health, and ECM degradation is one of the most prevalent pathological processes in IDD [40]. In IDD, MMPs and ADAMTSs induce and facilitate ECM degradation. With decreasing synthesis of significant collagen proteins including collagen II and cohesin, which constitute the internal NP, the IVD gradually loses its distinctive phenotypic features, anatomical structure, and function [41]. It has been reported that sesamin suppresses endotoxin-induced expression of MMP and ADAMTS in bone marrow cells [42]. We constructed an LPS-induced ATDC5 cell degeneration model and detected high MMP13 and ADAMTS5 protein expression, as well as low collagen II expression, and sesamin reversed the effect of LPS stimulation. Sesamin delayed the degradation of ECM. Furthermore, this study showed that LPS-induced degenerated cartilage cells had a high percentage of EdU positivity and low DNA replication, whereas the addition of sesamin increased DNA replication and promoted cell proliferation. Therefore, sesamin could alleviate IDD by promoting the proliferation of ATDC5 cells. These results showed that sesamin not only inhibited apoptosis but also delayed the degradation of ECM and promoted cell proliferation to treat IDD. Additionally, the multitarget nature of this drug therapy was realized.

There are limitations in this study. First, the available drug and disease databases are not comprehensive. We will continue to focus on new compounds and target genes to further improve the understanding of Naru 3 pill in the treatment of IDD. Second, the actions of the active compounds, targets and pathways selected in this study need further validation, and our next step is to further validate the potential mechanisms in vivo. Finally, this study did not investigate all predicted targets and pathways. Only apoptotic targets and pathways were selected for validation, and the others will be further validated, which is essential for a thorough understanding of Naru 3 pill.

## Conclusion

In summary, this study used a strategy combining network pharmacology, molecular docking, and experimental validation to explore the underlying mechanism of Naru 3 pill in the treatment of IDD. The network pharmacology analysis demonstrated that CASP3, Bax and BCL2 were essential target proteins, and apoptosis-related biological processes and apoptotic pathways were shown to play important roles. Combining in vitro experiments, molecular docking and molecular dynamics simulations, sesamin was shown to be the predominant active component in this therapeutic process. Subsequent studies confirmed that sesamin inhibited LPS-induced apoptosis and determined the expression of key targets CASP3, Bax and BCL2. In addition, sesamin delayed the degradation of ECM and promoted the proliferation of ATDC5 cells. It was shown that sesamin exerted a therapeutic effect on IDD by inhibiting apoptosis, promoting proliferation and delaying ECM degradation. In conclusion, this study preliminarily investigated the mechanism of Naru 3 pill in the treatment of IDD and shed new light on alternative herbal treatments.

## Supplementary Information

**Additional file 1: Table S1.** Sequences of mRNA associated with degeneration.

**Additional file 2: Table S2.** Antibodies used in this study.

## Abbreviations

IDD: Intervertebral disc degeneration
PPI: Protein‒protein interaction
GO: Gene ontology
KEGG: Kyoto Encyclopedia of Genes and Genomes
ECM: Extracellular matrix
LBP: Low back pain
IVD: Intervertebral disc
NP: Nucleus pulposus
AF: Annulus fibrosus
CEP: Cartilage endplate
TCM: Traditional Chinese Medicine
TMM: Traditional Mongolian medicine
OB: Oral bioavailability
DL: Drug-likeness
C-T: Component-target
C-T-D: Component-target-disease
BP: Biological process
CC: Cellular component
MF: Molecular function
RMSD: Root mean square deviation
LPS: Lipopolysaccharide
qRT‒PCR: Quantitative real-time polymerase chain reaction
WB: Western blot
PVDF: Polyvinylidene fluoride
CCK-8: Cell Counting Kit-8
