# Supplementary material for: The mechanism by which Naru 3 pill protects against intervertebral disc cartilage endplate degeneration based on network pharmacology and experimental verification
Source: J Orthop Surg Res. 2023 Jul 31;18:552. doi: 10.1186/s13018-023-04014-x (PMC10388481; doi:10.1186/s13018-023-04014-x)
Supplement: Supplementary file 1 — Additional file 1: Table S1. Sequences of mRNA associated with degeneration. [file 13018_2023_4014_MOESM1_ESM.docx]

Supplementary table 1 Sequences of mRNA associated with degeneration

| Gene | Primer | Sequence (5'-3') | PCR Products |
| --- | --- | --- | --- |
| Mus β-actin | Forward | CACGATGGAGGGGCCGGACTCATC | 240bp |
|  | Reverse | TAAAGACCTCTATGCCAACACAGT |  |
| Mus MMP13 | Forward | AGCCCTATCCCTTGATGCCATTA | 293bp |
|  | Reverse | CGCTCAGTCTCTTCACCTCTTTT |  |
| Mus MMP3 | Forward | TGAAGGAGAGGCTGACATAA | 334bp |
|  | Reverse | TCCATAGAGGGACTGAATAC |  |
| Mus collagen II | Forward | AGCAAGAGCAAGGAAAAGAA | 134bp |
|  | Reverse | GTGGACAGTAGACGGAGGAA |  |
| Mus Acan | Forward | ATGAGAGAGGCGAATG | 268bp |
|  | Reverse | TGCTTGTAGGTGTTGG |  |
| Mus ADAMTS5 | Forward | CTGGATGTGACGGCATTATT | 190bp |
|  | Reverse | GTCTGGTCTTTGGCTTTGAA |  |
| Mus Col-X | Forward | TCTGCTGCTAATGTTCTTGA | 164bp |
|  | Reverse | GAATGCCTTGTTCTCCTCTT |  |
| Mus SOX9 | Forward | ATGAAGATGACCGACGAGCA | 196bp |
|  | Reverse | TGCACACGGGGAACTTATCT |  |
