# Supplementary material for: The mechanism by which Naru 3 pill protects against intervertebral disc cartilage endplate degeneration based on network pharmacology and experimental verification
Source: J Orthop Surg Res. 2023 Jul 31;18:552. doi: 10.1186/s13018-023-04014-x (PMC10388481; doi:10.1186/s13018-023-04014-x)
Supplement: Supplementary file 2 — Additional file 2: Table S2. Antibodies used in this study. [file 13018_2023_4014_MOESM2_ESM.docx]

Supplementary table 2 Antibodies used in this study

| Antibodies | Source |
| --- | --- |
| rabbit anti- ADAMTS5 | affiniity |
| rabbit anti-collagenII | bioss |
| rabbit anti-MMP13  rabbit anti- caspase3 | Proteintech  Bioss |
| mouse anti-Bax | Santa Cruz |
| mouse anti-Bcl-2 | Invitrogen |
| mouse anti-β-actin | boster |
| goat HRP-anti-rabbit IgG | boster |
| goat HRP-anti-mouse IgG | boster |
